# Evaluating the effect of unidirectional loading on the piezoresistive characteristics of carbon nanoparticles

**DOI:** 10.1038/s41598-024-59673-5

**Published:** 2024-04-22

**Authors:** Bruno Alderete, Frank Mücklich, Sebastian Suarez

**Affiliations:** Chair of Functional Materials, Saarland University, Campus D3.3, 66123 Saarbrücken, Germany

**Keywords:** Carbon black, Carbon nanohorns, Carbon nanotubes, Graphene nanoplatelets, Graphite flakes, Onion-like carbon, Carbon nanotubes and fullerenes, Graphene, Nanoparticles, Two-dimensional materials

## Abstract

The piezoresistive effect of materials can be adopted for a plethora of sensing applications, including force sensors, structural health monitoring, motion detection in fabrics and wearable, etc. Although metals are the most widely adopted material for sensors due to their reliability and affordability, they are significantly affected by temperature. This work examines the piezoresistive performance of carbon nanoparticle (CNP) bulk powders and discusses their potential applications based on strain-induced changes in their resistance and displacement. The experimental results are correlated with the characteristics of the nanoparticles, namely, dimensionality and structure. This report comprehensively characterizes the piezoresistive behavior of carbon black (CB), onion-like carbon (OLC), carbon nanohorns (CNH), carbon nanotubes (CNT), dispersed carbon nanotubes (CNT-D), graphite flakes (GF), and graphene nanoplatelets (GNP). The characterization includes assessment of the ohmic range, load-dependent electrical resistance and displacement tracking, a modified gauge factor for bulk powders, and morphological evaluation of the CNP. Two-dimensional nanostructures exhibit promising results for low loads due to their constant compression-to-displacement relationship. Additionally, GF could also be used for high load applications. OLC’s compression-to-displacement relationship fluctuates, however, for high load it tends to stabilize. CNH could be applicable for both low and high loading conditions since its compression-to-displacement relationship fluctuates in the mid-load range. CB and CNT show the most promising results, as demonstrated by their linear load-resistance curves (logarithmic scale) and constant compression-to-displacement relationship. The dispersion process for CNT is unnecessary, as smaller agglomerates cause fluctuations in their compression-to-displacement relationship with negligible influence on its electrical performance.

## Introduction

The piezoelectric effect is the phenomenon of electrical charge concentration in materials when a force is applied^1^. This effect finds a plethora of applications since it can be exploited for sensors and actuators. Similarly, the piezoresistive effect is the change in a material’s electrical resistance caused by its dimensional changes, which can originate from external forces or thermal expansion^2^. Piezoresistance has a wide range of applications, and recent innovations in materials science have expanded the potential uses of piezoresistive sensors. These include structural health monitoring, motion detection in fabrics and wearables, and human–machine interfaces, among others^3,4^. The most commonly used materials for their piezoresistive behavior are metals and metal alloys, semiconductors, piezoceramics (such as perovskite and tetragonal/rhombohedral crystals), and conductive polymeric composites. However, as previously alluded, the piezoresistance behavior of these materials is highly dependent on temperature, thus requiring temperature compensation^3^. Carbon-based strain sensors are particularly attractive due to their temperature independence, which is a substantial advantage over conventional piezoresistive materials. This eliminates the need for additional dedicated electronics to compensate for thermal impact. The thermal stability of carbon nanoparticles (CNP) is not only sought after because they eliminate this requirement, but they can a withstand extreme temperatures (high and low), as well as thermal fluctuations^4^. This characteristic makes CNP a promising material for sensor applications.

Multiple researchers have studied the piezoresistive performance of CNP, however, their studies focus on individual nanostructures or incorporate CNP within composite materials^4–14^. However, there is a lack of literature analyzing the behavior and performance of CNP bulk powders. Due to the versatility of the carbon atom, it is capable of forming different carbon–carbon bonds. Thus, carbon can form different allotropes, which depending on the hybridization state, changes their properties^15,16^. Accordingly, in this study we sought out to evaluate the piezoresistive behavior of different carbon archetypical structures: i.e., quasi-zero-dimensional (fullerene-type), one-dimensional (nanotube-type), and two-dimensional (graphene-type) nanostructures. Therefore, the CNP evaluated in this study are carbon black (CB), onion-like carbon (OLC), carbon nanohorns (CNH), carbon nanotubes (CNT), graphite flakes (GF), and graphene nanoplatelets (GNP). Certain CNP tend to form agglomerates due to Van der Waals interactions. Therefore, to evaluate the effect of CNP agglomeration on its piezoresistance, the as-received CNT were dispersed to reduce the size of CNT agglomerates (henceforth CNT-D).

The electrical properties of CNP have garnered much attention from the research community, leading to a profusion of applications for these unique nanostructures. Applications range from the aforementioned sensing and actuating purposes to applications such as printed electronics and their incorporation as reinforcement phase in polymeric matrices^17–19^. Additionally, the transport properties of CNP make them suitable for electronic bridging and as terminal interconnection media in micro- and nano-electromechanical systems. Moreover, the piezoresistive behavior of CNP can be utilized to determine the effective contact load in electrical connectors. This is typically calculated through simplified modelling (e.g., finite element method). By placing a CNP interlayer between two connectors the contact load can be rapidly and easily determined by measuring the contact resistance (using Holm’s contact resistance equation). Therefore, understanding the piezoresistive behavior and determining the electrical resistance of CNP is of considerable interest in the field of sensors, electronics, and materials science.

In our previous work, different CNP have been used as protective coatings due to their hydrophobic behavior^20,21^, as topography offsetting interfaces due to their elasticity^22^, as well as for wear protection^23–27^. Moreover, CNP-containing composites have been studied for tribological and electrical applications^28–33^. However, in these studies, electrical characterization of the coating material or reinforcement phase was always in conjunction with the substrate or matrix material, respectively. Therefore, it is of significant interest to understand the electrical performance solely of the CNP. Thereupon, the objective of this work is to analyze the piezoresistive performance of the CNP under unidirectional compressive stress across a wide loading range. The results will determine the repeatability and reliability of the CNP, as well as identify the loading ranges with the best performance. This will enable the selection of the appropriate CNP for specific applications – such as wearable technology, strain sensors, etc. The six CNP analyzed were selected based on their mechanical and electrical properties, their geometry, their versatility, and their potential to be used as a straightforward piezoresistive medium. A non-conductive vessel was designed to contain the CNP between two electrodes and electrically characterized by load-dependent and current-dependent electrical resistance measurements under atmospheric conditions. The former aims to evaluate the piezoresistive behavior of the CNP, whereas the latter the ohmicity of the CNP^34^. The gauge factor (*K* from Eq. 1)^2^ represents the ratio between the change in electrical resistance of a piezoresistive material and the mechanical strain that the piezoresistive material has undergone. However, the CNP in this work are mainly subjected to compaction and to a lesser extent to mechanical strain due to the porous nature of the bulk powders. Therefore, an analogous relationship (*K*_*b*_ factor) is proposed and discussed.

1 $$K=\frac{\Delta R/{R}_{0}}{\varepsilon } ,\quad where\, \varepsilon =\frac{\Delta X}{{X}_{0}}$$

This work aims to analyze the piezoresistive behavior of bulk powder CNP with varying dimensionality, morphology, and chemistry. The characteristics of the CNP are subsequently correlated with their piezoresistive behavior and an adapted gauge factor is proposed to better predict their performance. This analysis is crucial since there is limited literature available that evaluates the influence of external compression on the conductivity of bulk CNP powders^35–37^. This work expands on the literature by correlating the change in electrical resistance with the strain—i.e., CNP displacement—and nanostructure dimensionality and morphology. In addition, with the aforementioned modified gauge factor it is possible to estimate the piezoresistive performance of the CNP without the need for experimental measurements.

## Materials and methods

**Figure 1:**
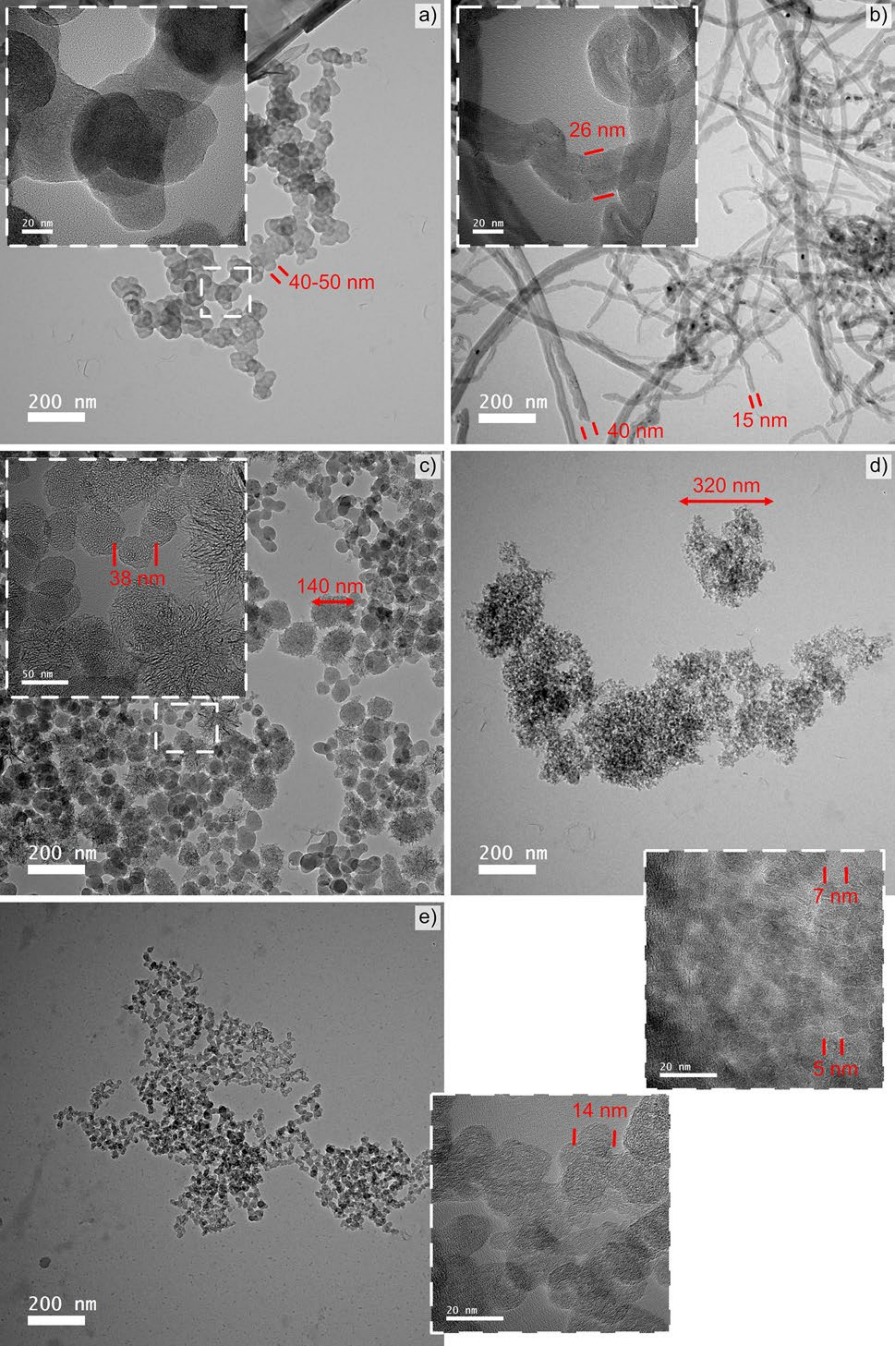
HR-TEM micrographs of (**a**) GNP, (**b**) CNT, (**c**) CNH, (**d**) OLC, and (**e**) CB acquired at ×50,000. Insets show magnified view of the different CNP.

In this study, six different CNP were analyzed (CB, OLC, CNH, CNT, GF, and GNP). The CB particles (Orion, Luxembourg) possess over 96% carbon, 0.2–1.3 wt.% hydrogen, 0.2–0.5 wt.% oxygen, 0–0.7 wt.% nitrogen, and 0.1–1 wt.% sulfur, with a mean particle size between 10 nm up to 500 nm, approximately. The OLC were synthesized from carbon nanodiamonds (NaBond Technologies Co., China),—carbon purity greater than 98%, with individual diameters ranging from 4 to 8 nm—by annealing the nanodiamonds in a graphite crucible in a vacuum furnace (between 1 × 10^−4^ and 1 × 10^−3^ mbar) with a tungsten heater (three hour holding time at 1700 °C with a heating rate of 10 °C)^38^. The single-walled CNH used were dahlia-type, produced by rapid condensation of carbon atoms without a catalyst (Carbonium SRL, Italy). The as-received high-purity CNH have a horn diameter between 3 and 5 nm, horn lengths between 30 and 50 nm, and a cluster diameter between 60 and 120 nm. The CNT used were chemical vapor deposition-grown multi-walled CNT (Graphene Supermarket, USA), with an outer diameter distribution between 50 and 85 nm, an as-received state length from 10 to 15 µm, and carbon purity over 94%. Dispersed CNT were analyzed with the aim of evaluating the influence of agglomerate size on the piezoresistive behavior of CNT. CNT agglomerate size was reduced by dispersing the as-received CNT in ethylene glycol (EG) at a concentration of 0.2 mg_CNT_/ml_EG_, followed by 5 min of homogenization (Ultra-Turrax T-25, IKA, Germany) at 7.5 krpm and 10 min in an ultrasound bath. This colloid was then placed inside a ventilated furnace at 150 °C for 48 h. GF (Alfa Aesar, Germany) with a median size between 7 and 10 µm, 99.8% purity were used. The GNP used consist of 3–8 graphene monolayers, with lateral dimensions between 2 and 8 µm, a carbon purity greater than 98 wt.% and a moisture content below 1 wt.% (Graphene Supermarket, USA). For morphological analysis, the different CNP were micrographed by electron microscopy (Helios™ G4 PFIB CXe DualBeam™ Super, Thermo-Fisher Scientific, The Netherlands) using Through-the-Lens detectors at an acceleration voltage of 5 kV and a current of 0.1 nA. In addition, the as-received CNP were micrographed by high resolution transmission electron microscopy (HR-TEM) to better analyze their morphology and dimensionality. The HR-TEM (JEOL ARM200, Japan) micrographs were acquired at 200 kV and are shown in Fig. 1. As the figure shows, three distinct dimensionalities can be observed. CB, OLC, and CNH (Fig. 1c–e) are quasi-zero-dimensional nanostructures, whereas the CNT (Fig. 1b) are one-dimensional nanostructures. Furthermore, GNP and GF (the latter of which cannot be micrographed by TEM due to the particle’s thickness) are two-dimensional carbon nanostructures. For the purposes of this study, and based on its performance, GF is considered a two-dimensional particle, despite it being dimensionally larger.

Electrical testing was performed using a custom tribo-electrical testing rig^39^. In order to measure the electrical resistance of the CNP, a few modifications were required, primarily the design of an appropriate vessel for the CNP. The vessel is a 20 mm long cylinder, with an outer diameter of 23 mm and an inner diameter of 12.7 mm. The cylinder is made of a non-conductive polymer. The ends of the vessel were capped with two aluminum disks; these disks are the contacting electrodes in the testing rig. The aluminum disks were ground (P1200 grit silicon carbide grinding paper) and polished (6 µm, 3 µm, 1 µm, and 0.25 µm) to obtain a mirror polished surface. After polishing, the disks were coated with Au_80_Pd_20_ via plasma-assisted PVD to reduce the influence of aluminum corrosion and to improve the electrical connection prior to electrical resistance measurements. The coating was performed using argon plasma at 2 × 10^−2^ mbar and a current between 20 and 25 mA. A total of 3 deposition cycles were carried out on all contacting electrodes, each of 2 min duration, for a total of 6 min. The roughness of the resulting coated aluminum caps was measured via confocal laser scanning microscopy (LEXT OLS4100, Olympus, Japan) and showed a root mean square roughness of S_rms_ = 0.03 µm. In the modified setup, one of the electrodes is mounted directly onto the testing rig. The other electrode is fixed onto the end of the plastic vessel via four grub screws. The electrode/vessel system was then mounted onto the sample holder of the testing rig using conductive double-sided adhesive copper foil (Plano GmbH, Germany).

**Figure 2:**
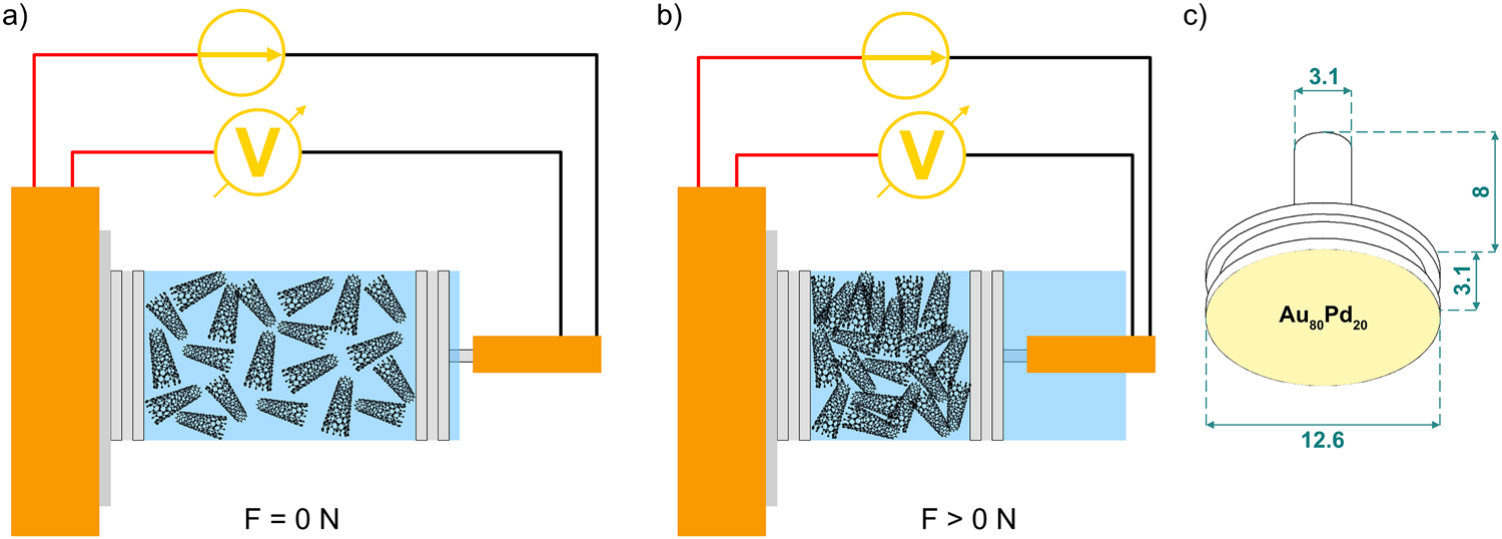
Schematic representation of piezoresistance measurements (**a**) prior to and (**b**) post compression. (**c**) Au_80_Pd_20_ coated aluminum vessel caps (electrodes).

For piezoresistance measurements, the vessels were filled with 100 mg of CNP. The setup is then mounted onto the testing rig for electrical characterization. A schematic representation of the electrical resistance measurements is shown in Fig. 2a, b. The uncompressed, porous nanoparticles are depicted in Fig. 2a, whereas Fig. 2b depicts the improved interconnectivity between the nanoparticles as the load increases. Figure 2c is a schematic of the aluminum vessel caps.

Piezoresistance of the CNP was measured via load-dependent electrical resistance between 0.25 N and 10 N (corresponding to pressures ranging from 2 kPa up to 80.3 kPa). At each load, a current of 0.05 mA_DC_ was supplied using a Keithley 2400 SMU source. The normal load was held constant during resistance measurements with an error consistently below 0.05 N (i.e., 0.4 kPa). The voltage drop between the parallel aluminum vessel caps was measured ten times per load using a Keithley 2182a nanovoltmeter. The displacement between each established load was measured using a high-precision linear stage (precision of 0.2 µm^39^) by acquiring the current position of the stage once each load was established. Further electrical analysis was carried out through current-dependent electrical resistance measurements (henceforth, current sweep) with the aim of evaluating the ohmic behavior—or lack thereof—of the CNP in question. Voltage drop was measured ten times and averaged at a constant normal load of 2 N (i.e., 16 kPa) sourcing currents between 1 µA_DC_ up to 10 mA_DC_. In both experiments, at least four tests were done per CNP with the bulk particles compacted prior to resistance measurements to achieve reliable and reproducible results. Therefore, the initial state of the CNP does not affect subsequent tests. During electrical tests, the ambient temperature and humidity were kept constant at 23 ± 2 °C and 57% ± 3% r.h., respectively.

Moreover, the dominant deformation mechanism that the CNP underwent was evaluated by the methodology proposed by Tamai et al.^40^. For this analysis, the load-dependent resistance curves were plotted in double logarithmic scale and the resistance curves linearly fitted. The slope of the fitted curve corresponds to the *n* factor in Eq. (2), where *R*_*t*_ is the total resistance, *F* is the force applied, and the numerator is a constant value that incorporates resistivity and hardness of the materials in question, as well as the influence of any surface contaminants present^41^.

2 $${R}_{t}={\left(\frac{Constant}{F}\right)}^{n}$$

When the exponent (slope of the fitted line) takes a value of − 1/3 or − 2/3 the methodology suggests that elastic deformation is predominant in the system. Conversely, if the *n* factor takes a value of − 1/2 or − 1, plastic deformation is dominant. Similarly, the methodology proposed by Tamai et al. can also be used to predict the main source of electrical resistance in the system. When the *n* factor takes a value of − 1/3 or − 1/2, the constriction resistance is dominant in the system, whereas film resistance dominates for values of − 2/3 or − 1. It is important to note, however, that this analysis is highly theoretical in nature and applicable to a single deforming body. The purpose of this methodology is to verify the applicability of the method and validate the elasticity of the CNP.

## Results and discussions

### Ohmic response

**Figure 3:**
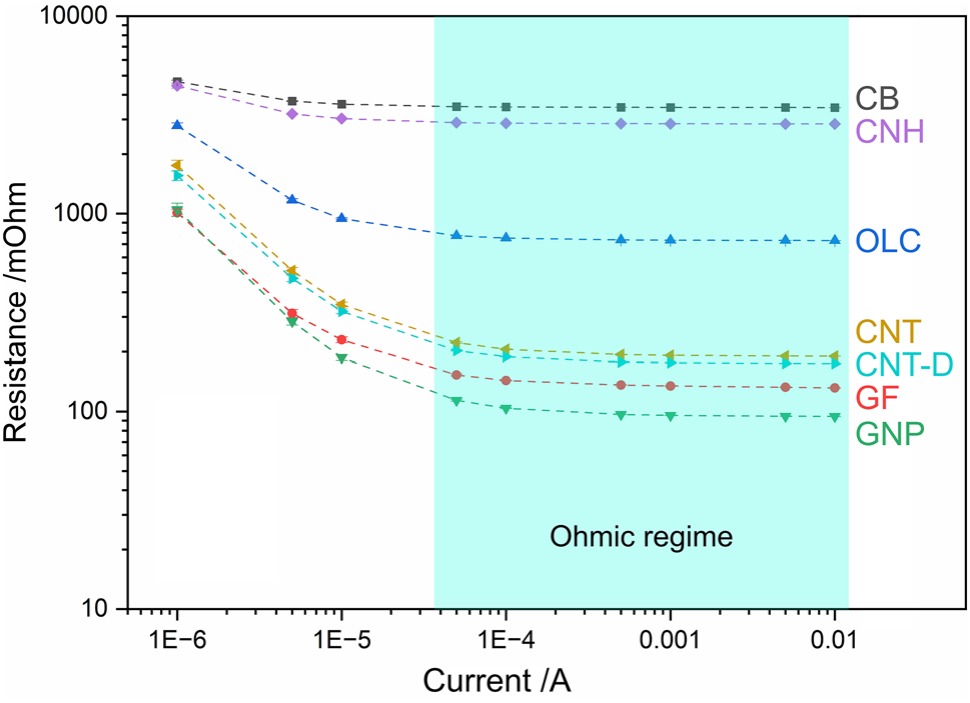
Results from current sweep of CNP at 2 N (i.e., 16 kPa). The shaded region from 0.05 to 10 mA highlights the ohmic regime.

The ohmic behavior of the different CNP was evaluated by performing current sweep tests. This experiment consists of contacting at a constant normal load and measuring the change in electrical resistance as different current levels are applied, thus providing information on the direct proportionality between the sourced current and the voltage drop measured. The results from the current sweep for the different CNP are shown in Fig. 3. The shaded region in Fig. 3 highlights the ohmic regime of the CNP, which is largely consistent for all nanoparticles from 0.05 mA to 10 mA. At lower current levels the resistance tends to increase, although the increase in resistance for currents below 0.05 mA depends on the particular CNP. The current is flowing through the CNP; therefore, the percolation path is established. However, the high resistance values observed for the CNP at low currents suggest that there is significant tortuosity in the system. Tortuosity refers to the ease or difficulty with which transport, in this case electrons, take place in a porous medium. The high tortuosity within the vessel is a consequence of the relatively low normal loads used, which inadequately compacts the CNP. Insufficient compaction results in a more complex conduction path, thus increasing tortuosity and electrical resistance. However, at the same normal load (2 N), higher currents facilitate electron conduction through the porous media without requiring additional compression to fill the voids. At lower current levels, one- and two-dimensional nanostructures (i.e., CNT, CNT-D, GF, and GNP) exhibit a significant increase of approximately one order of magnitude, whereas CB and CNH maintain consistent values throughout the entire range of currents analyzed. OLC displays an intermediate behavior, with resistance increasing below 0.05 mA, albeit to a lesser extent than one- and two-dimensional nanostructures. In other words, CB and CNH show a resistance difference of 35% and 56% respectively between 0.001 mA and 10 mA, OLC a difference of 281%, whereas CNT, CNT-D, GF, and GNP show a difference of 823%, 792%, 668%, and 1010%, respectively. This difference is likely due to a higher potential barrier caused by the poor arrangement of the particles at 2 N^42,43^. The insufficient compaction implies that voids are not filled and causes a reduction in the contact zones. Specifically for the case of OLC, due to their synthesis method (thermal annealing of nanodiamond), a non-conductive sp^3^ core remains, which could influence their transport^44,45^.

Observing the ohmic region of the CNP, at a constant normal load of 2 N (i.e., 16 kPa), the CNP arrange themselves in a clear hierarchical order. The two CNP with the highest ohmicity (CB and CNH) also exhibit the highest absolute resistance, with values ranging between 4,000 and 5,000 mΩ. In contrast, OLC shows a narrower ohmic range with an approximate resistance value of 700 mΩ. One- and two-dimensional nanostructures, on the other hand, show a similar ohmic range to OLC. However, these CNP show resistance values below 200 mΩ. The high absolute resistance of CB, CNH, and OLC can be associated with the morphology of the nanoparticles. Both CB and OLC nanoparticles can be classified as quasi-zero-dimensional nanostructures (refer to Fig. 1d, e). The former is a combination of fullerenic, amorphous, and graphitic carbon^46^, whereas the latter is comprised of concentric fullerenes with increasing diameters^47^. The classification of CNH is more difficult. Due to their closed cage structure, they can be classified as a subset of fullerenes with a high aspect ratio, or as a short single-walled CNT because of the similar chemistry between the two nanoparticles, as well as their elongated shape^48^. However, CNHs’ behavior during current sweep tests aligns more closely with zero-dimensional nanostructures.

The nanostructures with the lowest absolute resistance (i.e., CNT, CNT-D, GF, and GNP) are not fullerene-type nanostructures, but rather one- and two-dimensional. The results from the current sweep tests indicate that the two-dimensional structures have lower electrical resistance than one-dimensional (further discussed in “Appraisal of all CNPs’ piezoresistance”). Few-layer graphene exhibits the lowest resistance (below 100 mΩ), whereas as-received CNT show approximately twice the resistance in their ohmic regime. Comparing the ohmic response and absolute resistance values of as-received and dispersed CNT does not show significant differences. Therefore, for the purpose of this study, CNT agglomerate size is not as significant as initially predicted regarding CNTs’ transport characteristics.

The lack of ohmicity by the two-dimensional nanostructures (i.e., GF and GNP) in the low current range can be explained by the anisotropic behavior of these particles. The in-plane (basal plane, or *ab* direction) conductivity of GF and GNP is orders of magnitude higher than in the transversal direction (*c* direction)^49^. Accordingly, considering the movement of electrons within the vessel between the aluminum caps, it is highly desirable that the GF and GNP particles align themselves perpendicular to the surface of the aluminum caps rather than in a parallel manner. However, as will be discussed in “Piezoresistance”, the GF and GNP arrange themselves in an aleatory fashion within the vessel; nonetheless, after a certain load is reached, the flat nanoparticles take a predominant orientation which is transverse to the electron flow. GNP, in particular, tend to arrange themselves for the most part in a parallel fashion since the nanoplatelets lack the mechanical rigidity that GF presents. Consequently, for low currents, a capacitive effect is dominant in GNP, and to a lesser extent in GF. Thereupon, the equivalent circuit is not purely resistive, but rather a combination of resistive-capacitive (R–C circuit). The capacitive component in GNP and GF arises from the distance between individual graphene layers, causing a potential barrier^42,43^. As shown in Fig. 3, this potential barrier is more significant for lower currents. Since this work pertains to direct current, the capacitive component of the two-dimensional CNP functions as an open circuit. As the current increases, the electrical resistance of these CNP decreases without the need for further compaction due to the reduced relevance of the dielectric (i.e., gap between graphene layers). It is worth noting that graphene and graphite are metal-like (zero band gap) conductors. Therefore, the ohmic behavior for current above 0.05 mA is observed in Fig. 3 as the capacitance takes a less dominant role (due to higher currents enabling the potential barrier to be overcome). The conductive properties of CNT and CNT-D, on the other hand, are highly reliant on the chirality of the tubes. Statistically, multi-walled CNT—such as the ones used in this study—always have at least one tube that behaves as a metallic conductor^50^. Therefore, the absence of ohmic behavior in CNT and CNT-D can be attributed to their semiconductive properties at low currents, rather than a capacitive effect, as is the case with GF and GNP. As the current levels increase, the energy required for electrons to move towards a metallic tube within the same multi-walled CNT—i.e., interlayer potential barrier—becomes more easily available thus shifting from a semiconductive dominant system towards a metallic-conductive system. Therefore, as the current increases, the individual CNT’s resistance drops and shows an ohmic behavior. The interlayer potential barrier of multi-walled CNT is approximately 10 meV^51^. Therefore, at room temperature, where approximately 25 meV of thermal energy is available, interlayer coupling should be generated. Nonetheless, the presence of CNT agglomerates in CNT and CNT-D may increase the potential barrier, necessitating additional energy input (i.e., higher currents) to overcome it.

### Piezoresistance

The piezoresistive behavior of the CNP was analyzed by measuring their electrical resistance under different loads. During the test, the nanoparticle powders were compacted by the electrodes while the voltage drop between them was measured; thus, recording the electrical resistance as a function of compaction (i.e., normal load). To minimize uncertainty in the measurements and reduce instrumental error, load-dependent electrical resistance tests were divided into two sets of loads: low loads (red plot) ranging from 0.25 N up to 2 N and high loads (black plot) ranging from 2 N up to 10 N. The current used for the measurements was 0.05 mA, ensuring that the resistance values are within the ohmic regime of the CNP tested (as shown in Fig. 3). This is not trivial since this indicates that Holm’s contact resistance equation (${R}_{C}=\rho /2a$) is valid^52^. In other words, the resistivity in the system can be estimated based on the area of a flat circular contact of radius *a* and the electrical resistance measured. Due to the ohmic behavior of the system at 0.05 mA and the relatively large pressure applied on the CNP, the Sharvin resistance (${R}_{S}=C/{{a}_{c}}^{2}$) plays a minor role in the resistance measured^53^, where *a*_*c*_ is the constriction radius and the constant *C* depends solely on the electronic properties of the conductor. The reported resistance values correspond to the sum of the Holm (Drude diffusion^54^) and Sharvin (Knudsen diffusion^55^) resistance. However, the main contribution is from the former.

This section discusses the load-resistance curves and CNP displacement. It is important to note that the y-axis in the load-resistance curves is plotted on a logarithmic scale. Therefore, when referring to linear behavior this should be taken into consideration. The repeatability, stability, and low error of the resistance values at different normal loads is highlighted in Figs. S1, S2, and S3. These figures display four electrical resistance measurements for quasi-zero-dimensional, one-dimensional, and two-dimensional nanostructures, respectively.

#### Carbon black

**Figure 4:**
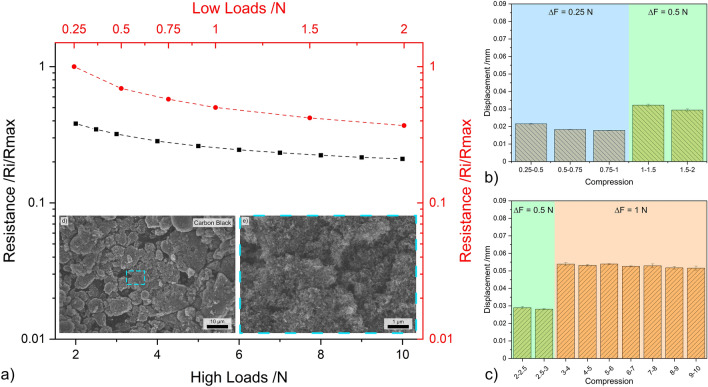
(**a**) Load dependent electrical resistance at low and high loads of CB. (**b**) and (**c**) Show the displacement for different compression levels at low and high loads, respectively. (**d,e**) SEM micrographs of CB (inset in load-resistance curves).

The results from load dependent electrical resistance for CB are shown in Fig. 4. Irrespective of the loading range, CB exhibits a relatively linear relationship between the applied load and the resistance—see Fig. 4a. In the lower loading range, CB exhibits the largest reduction in resistance. At 2 N the resistance of this nanostructure is only approximately 40% of the resistance measured at 0.25 N. However, at 10 N, CB’s resistance further decreases to approximately 20% of the resistance recorded at 0.25 N. The scanning electron microscope (SEM) micrograph shown in Fig. 4d highlights considerable CB agglomeration, with the magnified micrograph (Fig. 4e) depicting porous networks caused by the clustering of agglomerates. This supports the electrical performance observed in Fig. 4a. In the low loading range, most of the voids formed by large adjacent CB agglomerates are closed, thus causing a considerable decrease in resistance during initial compaction. This behavior is further supported by the TEM micrograph (see Fig. 1e), which shows that the agglomerates formed by CB are not highly compact. In the high loading range, however, increasing the normal load is not as effective at reducing resistance since now the multiple conductive paths have been established within the vessel due to CB and CB agglomerate compaction.

The bar graphs shown in Fig. 4b, c depict the displacement recorded in the three load increments analyzed, which were 0.25 N and 0.5 N in the lower loading ranges and 0.5 N and 1 N in the higher loading ranges. These two graphs demonstrate the reliability of CB in terms of the compression-to-displacement relationship. In all three load increments, the displacement remains relatively constant. For an increment of 0.25 N, the CB mass within the vessel was displaced by approximately 0.02 mm. The displacement value increased to approximately 0.03 mm and 0.05 mm for increments of 0.5 N and 1 N, respectively. The initial slight variations in displacement observed are supported by the TEM and SEM micrographs (Figs. 1e and 4d, e) since the compaction of large voids is required before the initial loads can be established and maintained. Therefore, CB demonstrates promising results due to its consistency in its compression-to-displacement relationship in the mid-to-high loading range after the voids formed due to agglomeration are filled, showing slight fluctuations at low loads.

#### Onion-like carbon

**Figure 5:**
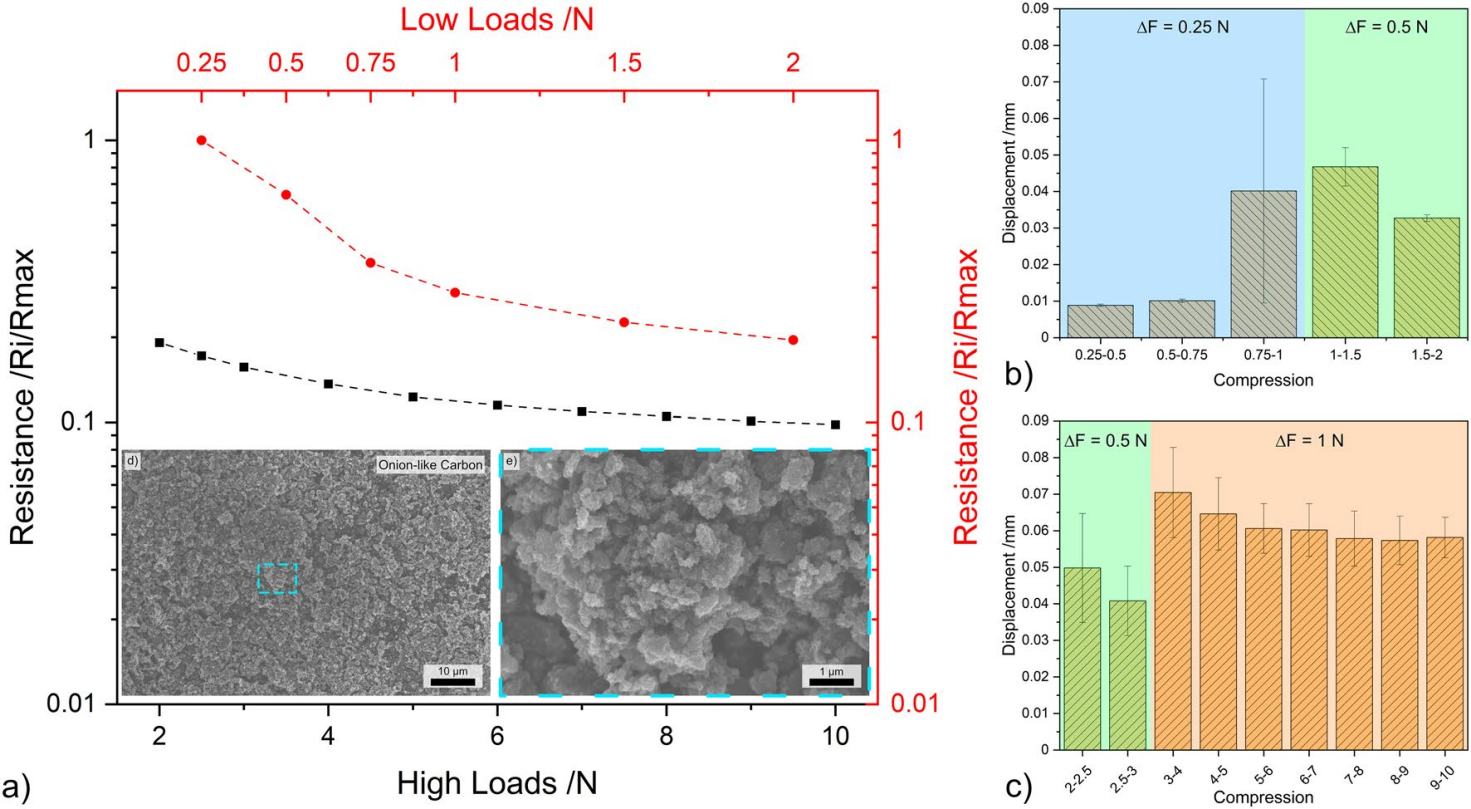
(**a**) Load dependent electrical resistance at low and high loads of OLC. (**b**) and (**c**) The displacement for different compression levels at low and high loads, respectively. (**d,e**) SEM micrographs of OLC (inset in load-resistance curves).

The piezoresistance curve for OLC in the lower loading range, shown in Fig. 5a, exhibits behavior similar to that observed in CB, with a sharp reduction in resistance as the load increases. In the high loading range, the resistance decreases as the normal load increases, but to a lesser extent. This similarity in performance is a consequence of the porous network observed in the micrographs—see Fig. 5d, e. The spherical morphology of OLC tends to favor the formation of voids between adjacent agglomerates as demonstrated in the magnified micrograph (Fig. 5e). Consequently, as the load increases in the lower loading range the particles accommodate the deformation by moving into the voids, thus filling them, and thus increasing connectivity. The SEM micrographs highlight the tendency of the OLC to form agglomerates in the µm-range. However, recalling the HR-TEM micrograph (Fig. 1d) OLC bulk powders form a more massive aggregate than the CB powder (Fig. 1e), with the latter showing more scattered CB agglomerates rather than a cohesive mass. This is a key distinction between OLC and CB, which affects the piezoresistive performance of the bulk powders. Due to OLC’s morphology and cohesive behavior, these nanostructures tend to show high elasticity. Therefore, the considerably lower resistance values measured are not only a consequence of void compaction, but also due to the compression of the OLC themselves. Furthermore, the magnified TEM micrograph in Fig. 1d illustrates the enhanced inter-particle connectivity within the OLC agglomerates. This improved interconnectivity accounts for the 20% resistance observed at 2 N and the 10% resistance observed at 10 N, both in comparison to the resistance at 0.25 N. It is worth noting that while CB and OLC are both quasi-zero-dimensional carbon nanostructures, the differences in their piezoresistive behavior is significantly influenced by intra-particle interactions and particle interactions within the powder’s agglomerates.

The compression-to-displacement relationship in OLC is not linear, showing multiple fluctuations within the same load increments—see Fig. 5b, c. The displacement varies from approximately 0.01 mm to 0.04 mm at increments of 0.25 N, and fluctuates between 0.03  and 0.05 mm at increments of 0.5 N. For increments of 1 N, the displacement stabilizes above 6 N at 0.06 mm per 1 N. The variability in compression-to-displacement highlights the elasticity of OLC. The observed variations between 0.75 and 3 N suggest the onset of particle accommodation. Below 0.75 N, the displacement is caused by compaction and filling of the voids, whereas for loads above 3 N the displacement is due to the elastic deformation of OLC. Between 0.75 and 3 N, the compression-to-displacement results indicate a hybrid state where the displacement is caused by OLC adjustment and where elastic deformation begins. Therefore, compaction of the voids does not play such a crucial role, but rather the compression of the concentric fullerenes at higher loads. This behavior is desirable for electrical performance since it improves interconnectivity when compacted. However, the use of this nanoparticle for its piezoresistive performance must be limited to high load applications, preferably above 6 N. This is because, for lower loads, obtaining consistent and reproducible piezoresistive measurements is difficult (see Fig. S1). This inconsistency hinders the tracking of particle displacement, or normal load applied, by directly measuring the electrical resistance of the resistance, and vice versa.

#### Carbon nanohorns

**Figure 6:**
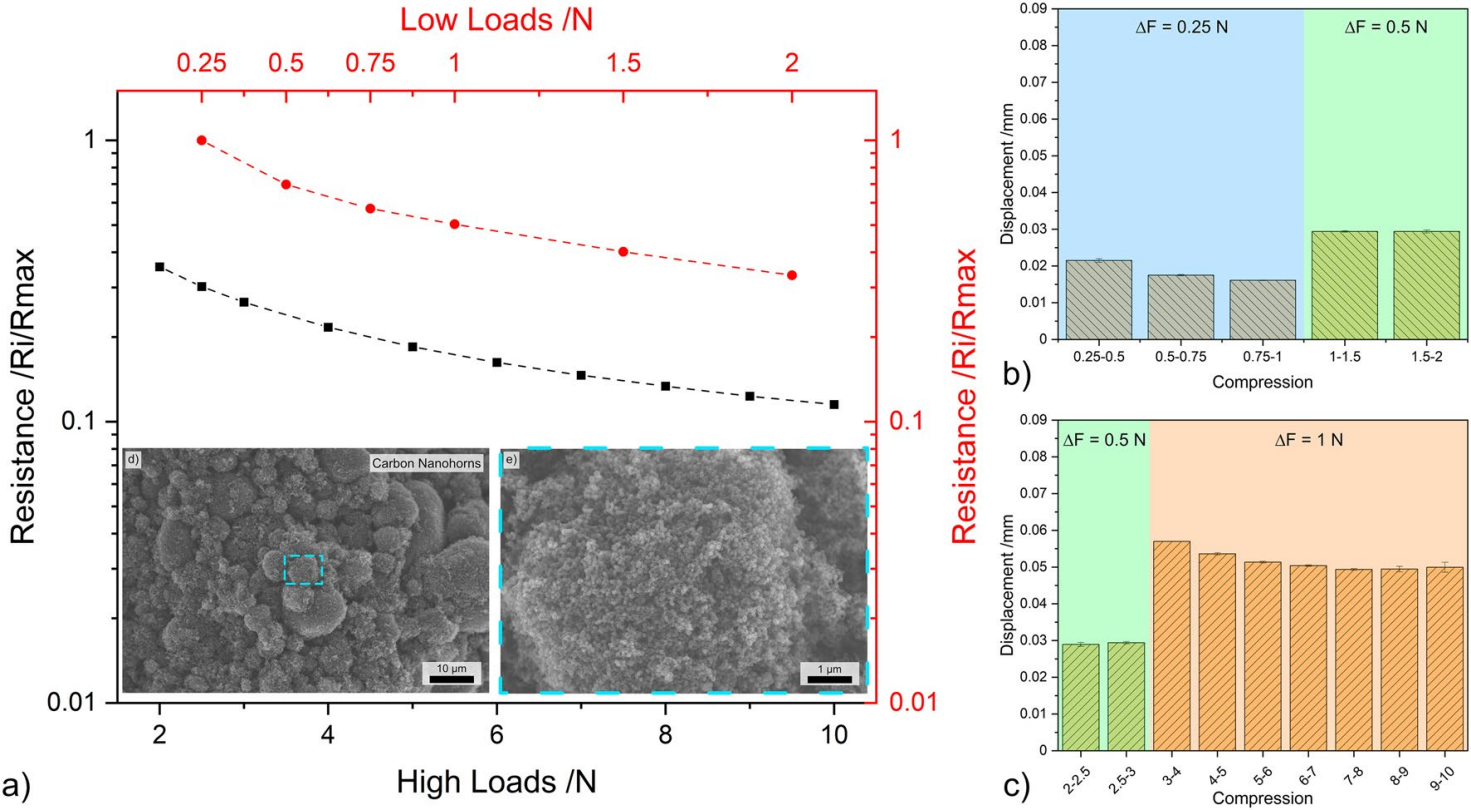
(**a**) Load dependent electrical resistance at low and high loads of CNH. (**b,c**) Show the displacement for different compression levels at low and high loads, respectively. (**d,e**) SEM micrographs of CNH (inset in load-resistance curves).

CNH exhibit exceptional linearity in both low and high loading ranges, as shown in Fig. 6a. This performance is highly desirable in applications that utilize piezoresistance as a working principle. The resistance at 2 N is approximately 35% of the initial resistance, decreasing to 10% at 10 N. The micrographs in Fig. 6d, e reveal that the CNH form large agglomerates, which create a complex porous network within the vessel. The TEM micrograph shown in Fig. 1c, reveals that the size of the CNH agglomerates are significantly smaller than those observed for the other zero-dimensional nanostructures. As a result, the agglomerates can adjust and compact as the load increases, making it easier to fill the voids. However, the consistency in the load-resistance curves is due to the remarkable elasticity of the nanohorns. This elastic behavior of the nanohorns can be attributed to the CNT-like horns, which are capable of deforming under compressive loads, thus inverting the cone from tip to base^48^. Therefore, after the CNH have filled all voids resulting from compressive loads, the cone structures absorb the energy through mechanical deformation. This improves particle interconnectivity and enhances the conductive paths for electron mobility.

The compression-to-displacement relationship (Fig. 6b, c) indicates consistent results. Minor displacement variations were observed for increments of 0.25 N, but the value stabilizes after initial voids are closed. For increments of 1 N, the CNH bulk within the vessel is displaced by approximately 0.05 mm. This value was achieved for loads above 5 N; below 5 N, the displacement approaches 0.06 mm. For increments of 0.25 N and 0.5 N the displacement is approximately 0.02 mm and 0.03 mm, respectively. Thus, CNH demonstrate a wide range of applicability. The load-resistance curve of these nanoparticles exhibits exceptional linearity, and their compression-to-displacement relationship yields consistent results for both low and high loads.

#### Carbon nanotubes

**Figure 7:**
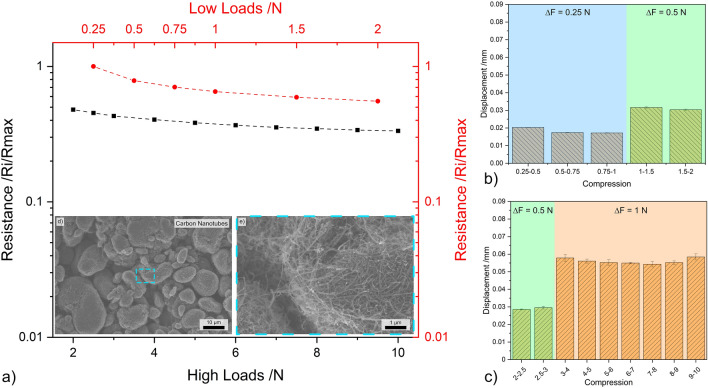
(**a**) Load dependent electrical resistance at low and high loads of CNT. (**b,c**) The displacement for different compression levels at low and high loads, respectively. (**d,e**) SEM micrographs of CNT (inset in load-resistance curves).

The CNT in their agglomerated state demonstrate a linear load-resistance relationship, as shown in Fig. 7a. Unlike the zero-dimensional nanostructures, there are no sharp reductions in electrical resistance. At 2 N, CNT show approximately 55% of the initial resistance, and the percentage drops to approximately 35% at 10 N, thus showing the smallest percentual reduction among the CNP analyzed. The electrical performance is not negatively impacted by the large voids formed due to CNT agglomerates, as shown in the micrograph in Fig. 7d. However, the magnified micrograph (Fig. 7e) reveals that individual CNT and small CNT bundles bridge the gaps between agglomerates, which is a distinctive characteristic of CNT. Therefore, in this case, the voids formed by two nanoparticle agglomerates do not significantly affect the electrical performance, as is the case in zero-dimensional nanoparticles (CB, OLC, and CNH). Additionally, the lack of a sharp reduction in the load-resistance curves indicates that the maximum inter-tube distance for tunneling (1.8 nm) was already achieved at 0.25 N^42^. To achieve a distance of 1.8 nm, low compression is necessary. This is because the equilibrium distance between two single-walled CNT is 3.4 Å^56,57^. As a result, even at low loads, tunneling or coupling is activated between adjacent CNT, allowing electrons to move from one tube to another.

The compression-to-displacement relationship of CNT (shown in Fig. 7b, c) is similar to that of CB (Fig. 4b, c). There are almost no fluctuations within each interval. Initially, CNT are displaced by approximately 0.02 mm. This value increases to approximately 0.03 mm and 0.06 mm for 0.5 N and 1 N increments, respectively. Due to the abundant presence of CNT agglomerates, it was expected that considerable displacements would occur initially. However, the large network of agglomerate within the vessel causes large-scale voids which require compaction prior to establishing the initial load. Therefore, once a force 0.25 N is reached, most of the large voids within the vessel have already been filled by the compaction and rearrangement of CNT agglomerates. As a result, the resistance at 0.25 N is exceptionally low and does not exhibit any measurable abrupt changes. Furthermore, since most CNT are in their agglomerated form, the minimum distance for electron coupling is already fulfilled (within each agglomerate). Therefore, the external load is only necessary to establish a contact between adjacent agglomerates, which occurs at low loads.

#### Dispersed carbon nanotubes

**Figure 8:**
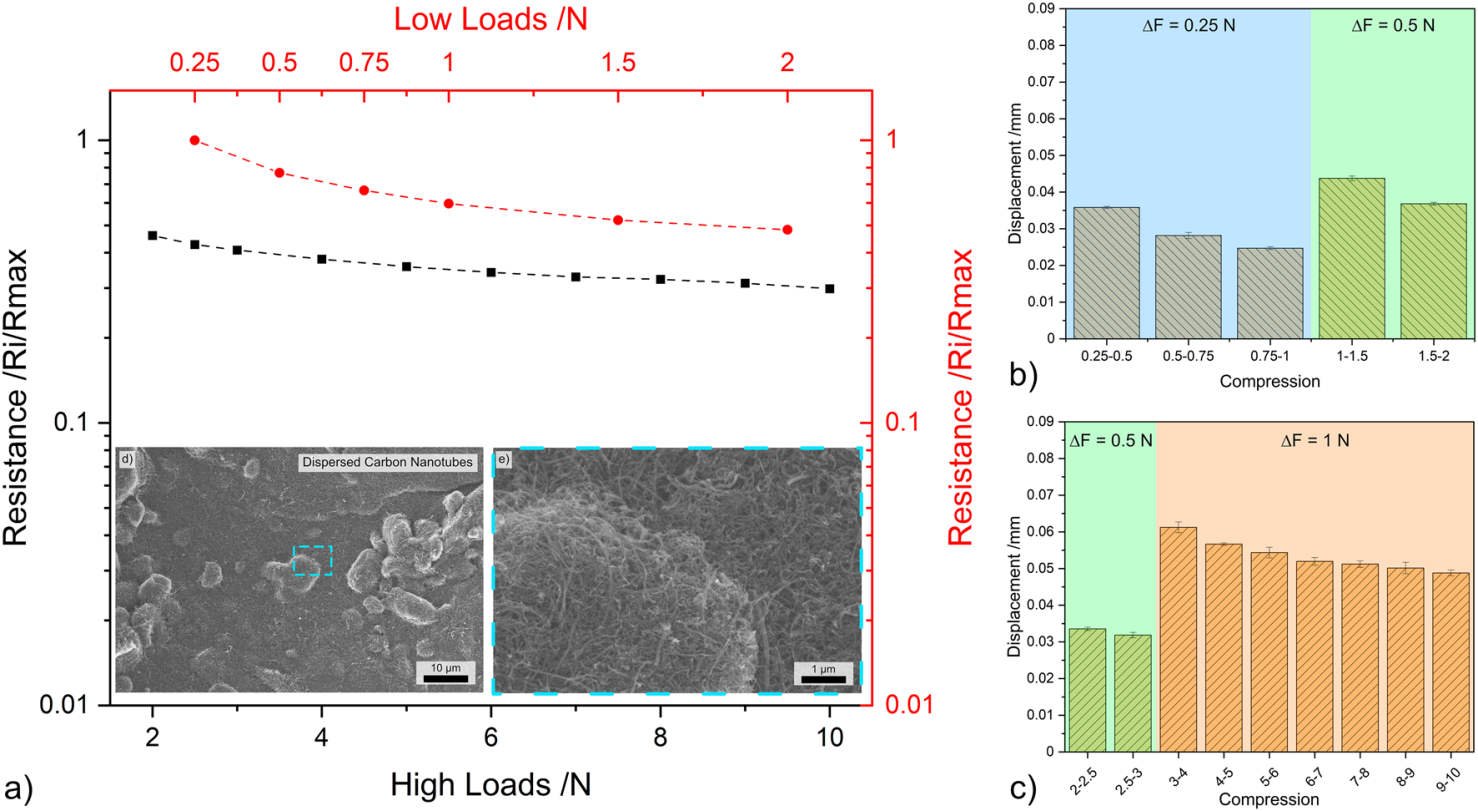
(**a**) Load dependent electrical resistance at low and high loads of CNT-D. (**b,c**) The displacement for different compression levels at low and high loads, respectively. (**d,e**) SEM micrographs of CNT-D (inset in load-resistance curves).

CNT have a strong tendency to form agglomerates due to Van der Waals interactions. Therefore, the as-received nanoparticles have a high degree of agglomeration which could negatively affect the piezoresistive performance of the nanoparticles. Therefore, it is important to analyze the influence of the agglomerate size on the piezoresistive behavior of the CNP since the electrostatic interactions within the agglomerates are significant. After dispersion, the CNT will re-agglomerate as the solvent evaporates. However, the overall agglomerate size will be smaller than in their as-received state^38,58^. The electrical performance of CNT-D is shown in Fig. 8a. As previously mentioned in “Ohmic response” (see Fig. 3), the similarity in electrical performance between CNT (Fig. 7a) and CNT-D (Fig. 8a) was not expected as it was hypothesized that the presence of larger CNT agglomerates would hinder the electrical performance of the as-received CNT. The resistance at 2 N and 10 N is approximately 50% and 30% of the initial resistance at 0.25 N, respectively. The micrographs (Fig. 8d, e) show that although the dispersion process was effective in breaking up large CNT agglomerates, multiple agglomerates remain, albeit to a much lesser extent (quasi-buckypaper). Prolonging the dispersion process would undoubtedly further minimize the presence of CNT agglomerates; however, prolonged sonication damages the structural integrity of the CNT^38,58^. Therefore, this is not a viable option as structural damage would reduce the desirable mechanical and transport properties of the CNT. However, despite the fact that CNT agglomerates are still found within the vessel, there is a large amount of disentangled CNT as shown in the micrographs.

The compression-to-displacement relationship for CNT-D (shown in Fig. 8b. c) is very different from that of CNT. In the as-received state, negligible fluctuations were observed between the different load increments. After dispersion, however, the compression-to-displacement relationship resembles that of CNH. In the 0.25 N and 0.5 N increments the displacement values tend to decrease, ranging from approximately 0.035 mm to 0.025 mm and 0.04 mm to 0.03 mm, respectively. The displacement measured at 1 N increment is initially high at approximately 0.06 mm and gradually decreases to about 0.05 mm. As previously discussed, the gradual reduction in the displacement as the normal load increases is due to particle elasticity. This behavior was also observed for the zero-dimensional particles (i.e., OLC, CNH, and to a lesser extent CB). Elastic behavior was more prominent in CNT-D since the compression acts upon small CNT clusters rather than on larger agglomerates. The compressive forces caused elastic deformation of the multi-walled CNT instead of the compacting of agglomerated CNT bundles. The displacement did not stabilize in the analyzed loading ranges, indicating that higher loads are necessary to establish a consistent compression-to-displacement relationship. Therefore, CNT-D is suitable for very high-load applications. However, for lower load applications, it is not necessary to reduce the size of agglomerate, since smaller agglomerates cause the fluctuation of the compression-to-displacement relationship and the piezoresistive behavior was not significantly affected by the presence of larger agglomerate sizes.

#### Graphite flakes

**Figure 9:**
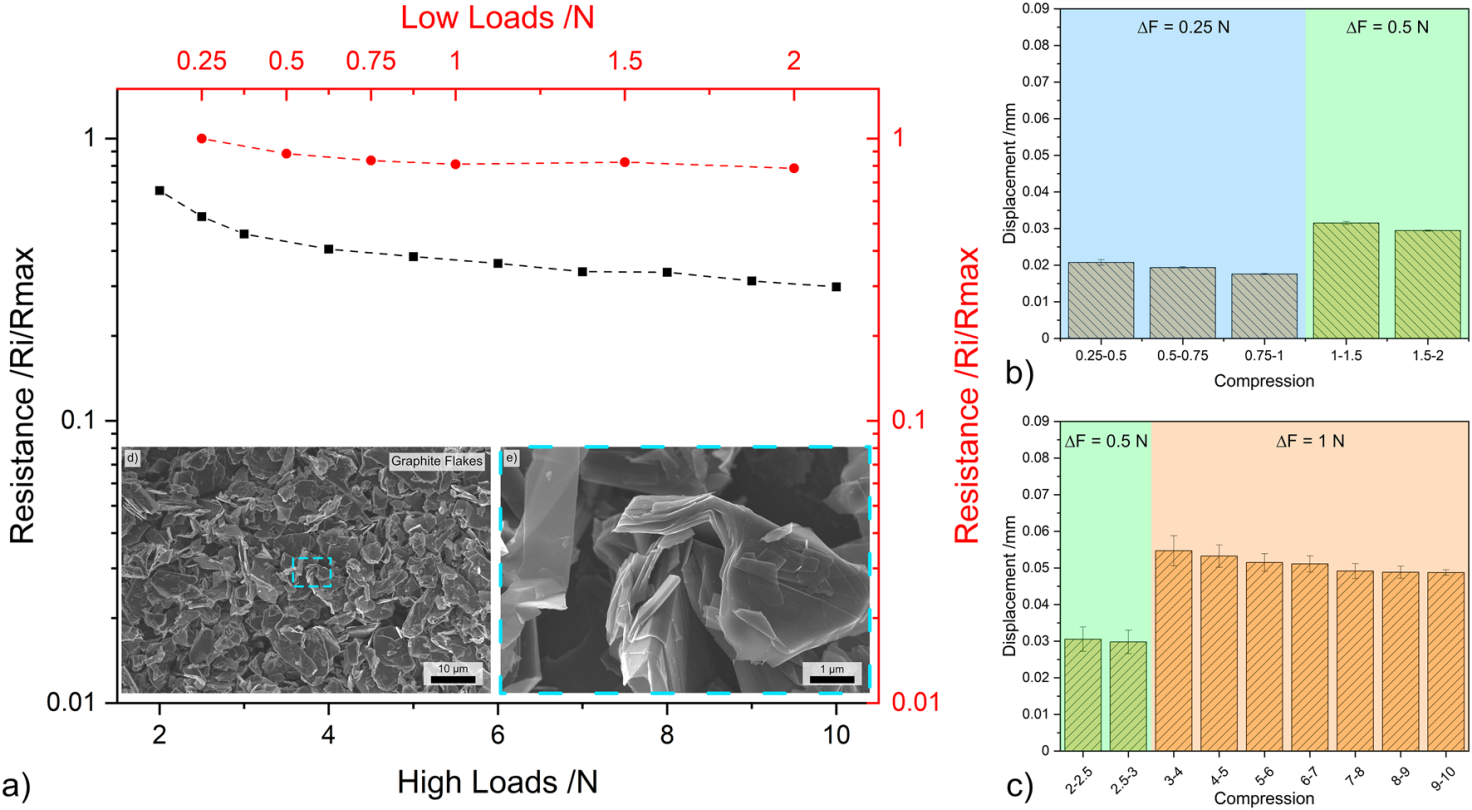
(**a**) Load dependent electrical resistance at low and high loads of GF. (**b,c**) The displacement for different compression levels at low and high loads, respectively. (**d,e**) SEM micrographs of GF (inset in load-resistance curves).

The load-resistance curves for GF (shown in Fig. 9a) contrast those observed for zero- and one-dimensional nanostructures in the fact that the resistance of GF does not decrease as significantly in the lower loading ranges, but rather in the higher ranges. At 2 N the resistance of GF decreases to approximately 80% the resistance at 0.25 N, with the value decreasing to about 30% at 10 N, thus showing the opposite behavior compared to zero-dimensional nanostructures (Figs. 4, 5, 6) where the predominant drop in resistance was recorded in the lower loading range. As opposed to zero- and one-dimensional nanostructures, GF does not form agglomerates. GF particles are large, with voids formed between adjacent flakes (see Fig. 9d, e). The micrographs demonstrate a certain degree of GF interconnectivity. However, the large and irregular shape of these particles requires higher loads to close the voids, which increases compaction within the vessel and improves flake interconnectivity. Additionally, the compaction of flakes oriented parallel to the surface of the electrodes promotes the reduction of the inter-graphene layer potential barrier. Reducing the potential barrier establishes more conduction paths, which in turn reduces the resistance of the bulk GF flakes by eliminating capacitive barriers—which act as open circuits since the tests were conducted with direct current.

Similarly to zero-dimensional nanostructures (excluding OLC) and as-received CNT, GF also exhibits relatively stable compression-to-displacement relationship (Fig. 9b, c), demonstrating the reproducibility of the recorded results (see Fig. S3). Due to the lack of GF agglomerates, the displacement recorded for load increments of 0.25 N is more consistent than that of zero- and one-dimensional nanostructures, at approximately 0.02 mm. Similarly, displacements of 0.03 mm were recorded at increments of 0.5 N. However, slight variations were observed at increments of 1 N, with displacement values of approximately 0.05 mm showing higher standard deviation. However, as the compression increases beyond 7 N, the error tends to decrease while the displacement values remain constant. This behavior is also supported by the load-resistance curves in the high loading range, which show linear load-resistance curves as the compression-to-displacement relationship remains constant.

#### Graphene nanoplatelets

**Figure 10:**
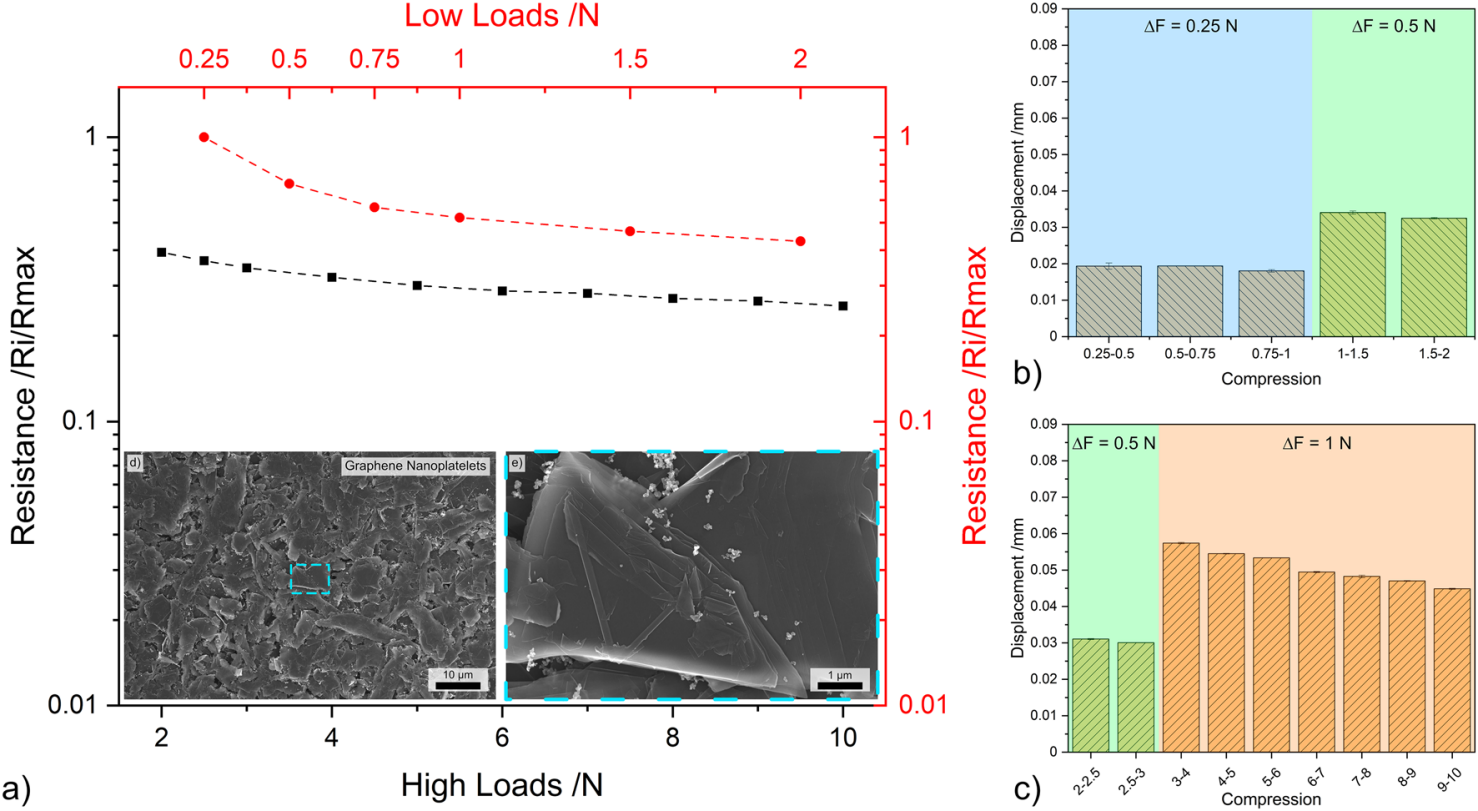
(**a**) Load dependent electrical resistance at low and high loads of GNP. (**b,c**) The displacement for different compression levels at low and high loads, respectively. (**d,e**) SEM micrographs of GNP (inset in load-resistance curves).

The piezoresistive behavior of GNP is shown in Fig. 10a. This nanoparticle shows a considerable reduction in resistance for the lower loading range, at 2 N reducing to approximately 40% the resistance measured at 0.25 N. In the higher loading range, it gradually reduces in a linear fashion to approximately 25% of the initial resistance (due to the rearrangement of the GNP—analogous to granular flow^59^), resembling the piezoresistive performance of one-dimensional nanostructures. As shown in Fig. 10d, e, the majority of the nanoplatelets align horizontally, in contrast to the observations in GF. This explains the higher initial reduction in resistance. The linear behavior in the higher loading range suggests that the voids have already been filled by GNP and the gradual reduction in resistance can be attributed to the compaction of individual nanoplatelets and nanoplatelet clusters. Furthermore, the thinner GNP are mechanically weaker than the thicker GF. Consequently, GNP require lower external pressure to orient the nanoplatelets parallel to the surfaces applying the load. Additionally, the micrographs illustrate individual nanoplatelets stacked in a disorderly manner, rather than GNP agglomerates. The magnified micrograph (Fig. 10e) reveals the presence of impurities (bright spots scattered throughout the nanoplatelet). These particles are likely a result of the synthesis process. Although the synthesis process details are unknown, it is reasonable to assume that the impurity particles are amorphous carbon. The impurities are deposited on the few-layer graphene platelets and are also found free-standing between them. However, the presence of amorphous carbon does not hinder the electrical performance of GNP, as it exhibits similar behavior to GF (see Fig. 9a).

The compression-to-displacement graphs (Fig. 10b, c) for GNP resemble those from GF in terms of values, but also in the fact that the displacement varies slightly for the load increments. For 0.25 N and 0.5 N increment the displacement is approximately 0.02 and 0.03 mm, respectively. The 1 N increment shows the most variation, ranging from approximately 0.06 mm down to 0.04 mm. Although the displacement values are not constant for the 1 N increments, the standard deviation in all measurements is negligible, thus suggesting the reproducibility of the results. In contrast to GF and OLC, GNPs’ displacement does not reach a stable value in the loading range analyzed, similarly to CNT-D. Therefore, the use of GNP for its piezoresistive behavior should be limited to low loads to ensure consistent results. GNP could be used for high load applications, however higher loads than those in the scope of this study are required.

### K_b_ factor

The gauge factor, as shown in Eq. (1), is a useful tool for evaluating the sensitivity of strain sensors. A high gauge factor indicates an increased output signal. However, by definition, it is not directly applicable to the systems evaluated in this study because it is not possible to measure the undeformed length and resistance of the bulk mass within the vessel. The gauge factor equation was adjusted in consideration of the fact that compressive stress is applied to an aggregate of nanostructures, rather than a single nanostructure (see Eq. 3). In the modified equation proposed, *R*_*x*_ is the resistance value measured, *R*_0.25_ is the initial resistance measured at 0.25 N, *∆D* is the displacement difference, *∆D*_*max*_ is the displacement difference between 0.25 N and 10 N, *∆F* is the load difference, and *F*_*max*_ is the maximum load. Therefore, the bulk powder gauge factor (henceforth *K*_*b*_) evaluates the linearity of the load-resistance curves (when plotted in a logarithmic scale), the consistency in the compression-to-displacement relationship and incorporates the loading range. The *K*_*b*_ factor values for each CNP are shown in Table 1 as the average for each load increment.

**Table 1:** *K*_*b*_ factor for all CNP based on the load increments.

CNP	0.25 N	0.5 N	1 N
CB	0.41 ± 0.02	0.33 ± 0.02	0.25 ± 0.02
OLC	2.18 ± 1.50	0.51 ± 0.11	0.42 ± 0.01
CNH	0.42 ± 0.01	0.29 ± 0.04	0.16 ± 0.02
CNT	0.56 ± 0.02	0.49 ± 0.05	0.38 ± 0.02
CNT-D	0.35 ± 0.02	0.38 ± 0.01	0.38 ± 0.03
GF	0.41 ± 0.01	0.40 ± 0.09	0.26 ± 0.01
GNP	0.42 ± 0.04	0.34 ± 0.02	0.30 ± 0.01

3 $${K}_{b}=\frac{{R}_{x}/{R}_{0.25}}{\Delta D/{\Delta D}_{max}}\cdot \frac{\Delta F}{{F}_{max}}$$

The *K*_*b*_ factor proposed for bulk powders under unidirectional compressive stress assesses the reproducibility of the piezoresistive performance of the powders. The *K*_*b*_ factor values indicate the poor performance of OLC for low loading ranges (i.e., 0.25 N increments). However, for mid-to-high loading ranges, the performance improves with slight variations not only in the *K*_*b*_ factor values, but also verified by the load-resistance curves and compression-to-displacement relationship (see Fig. 5). Moreover, the *K*_*b*_ factor for CB, CNH, CNT, and GNP decreases as the load increments increase, whereas GF remains constant for the first two load increments and CNT-D remain constant throughout. The fluctuations in *K*_*b*_ for the zero-dimensional nanostructures and CNT can be attributed to their tendency to form agglomerates. As a result, these structures must be displaced to fill the voids formed between agglomerates, which increases both the initial *∆D* and *∆D*_*max*_, causing the *K*_*b*_ factor to vary between loading increments. For GNP, on the other hand, the adjusting and subsequent stacking of the graphene structures may be responsible for larger initial displacements, which could affect the *K*_*b*_ factor values. In contrast, GF are more mechanically rigid, requiring higher loads for the rearrangement of the large flakes. As a result, the *K*_*b*_ factor values remain relatively constant for increments of 0.25 N and 0.5 N, whereas it slightly decreases for 1 N increments (see Fig. 9). The *K*_*b*_ factor for CNT-D remains exceptionally stable for the three load increments evaluated due to the low agglomerate state of the nanotubes. It should be noted that the *K*_*b*_ factor for CNT in the high loading ranges is the same as the values observed for CNT-D. This suggests that larger CNT agglomerates perform similarly to smaller agglomerates at sufficiently high loads. This validates that voids in bulk powders with larger CNT agglomerates were effectively compacted at higher loads, and further loading will have the same effect regardless of the agglomerate state of the CNT.

Therefore, the *K*_*b*_ factor can be used as a general guide to rapidly assess the reproducibility and reliability of a specific CNPs’ piezoresistive performance for different load increments. However, to comprehensively understand the behavior, it is crucial to analyze the load-resistance curves and the compression-to-displacement relationship.

### Appraisal of all CNPs’ piezoresistance

**Figure 11:**
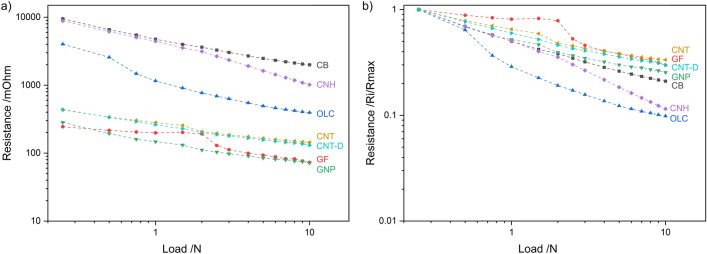
(**a**) Absolute and (**b**) normalized load dependent electrical resistance for the entire loading range. Note: the curves were plotted in double logarithmic scale.

The absolute and normalized load-dependent electrical resistance values for both loading ranges (plotted in double logarithmic scale to highlight the linear load-resistance relationship) are shown in Fig. 11. This plot summarizes and expands upon the individual discussions for each CNP. Throughout the entire loading range, the same hierarchical order and groupings observed in Fig. 3 are maintained. This ordering of the CNP coincides with the particles’ dimensionality, which dominates their quantum mechanical electronic properties. In other words, the electronic properties of the CNP are highly dependent on their electronic density of states^60^. As the dimensionality of the nanostructures changes, so does their electron distribution. Based on Fig. 11a and Fig. 3, it is evident that the zero-dimensional nanoparticles (CB, OLC, and CNH) exhibit the highest electrical resistance due to strict electron motion confinement in low dimensions due to quantum confinement effects, which results in a discrete free-electron density of states^60,61^. On the other hand, one-dimensional structures (CNT and CNT-D) display continuous spikes in free-electron density of states. The resistance observed for CNT and CNT-D in Fig. 11a is significantly lower than that observed for zero-dimensional nanostructures. In contrast, the two-dimensional structures (GF and GNP) have the highest free-electron density of states among the CNP analyzed. In this case, the staircase-like energy distribution more closely resembles the free-electron density of states of a bulk material, where the electron density of states is proportional to the square root of the energy. One-dimensional and two-dimensional structures, however, have anisotropic transport characteristics. Therefore, the electron density of states in the axial direction (in one-dimensional structures) and in the *ab* direction (in two-dimensional structures) varies significantly from the transversal direction (axial direction and *c* direction in one-dimensional and two-dimensional structures, respectively), thus affecting their transport characteristics.

Although two-dimensional structures exhibit a capacitive component due to the potential barrier between adjacent graphene layers, these nanoparticles demonstrate the lowest resistance. However, as discussed in “Ohmic response”and shown in Fig. 3, the current used in load-dependent resistance measurements (0.05 mA) is high enough to overcome the potential barrier. GF, in particular, exhibits a clear slope change before and after 2 N. The change may be caused by the rearrangement of vertical flakes; however, due to the mechanical rigidity of this structure, this is unlikely. The change is more likely due to the compaction of flakes that are parallel to the surface of the aluminum caps. In this case, as the load increases, the dielectric separation (interplanar distance in the *c* direction, which is 0.671 nm in the undeformed state^49^) decreases, minimizing the capacitive component’s relevance in the circuit. As a result, for sufficiently high normal loads and/or currents, the electrical circuit becomes purely resistive with a minimal capacitive component, following Ohm’s law (see Fig. 3).

After normalizing the data from Fig. 11a, a different hierarchy is established, as shown in Fig. 11b. The normalized values at 10 N highlight the overall change in resistance as the load increases. In this case, the one- and two-dimensional nanostructures, which exhibit the lowest absolute electrical resistance, also show the smallest resistance change from start to finish. After normalization, CB is grouped with the one- and two-dimensional nanostructures, despite having the highest absolute resistance. This quasi-zero-dimensional structure exhibits exceptional linearity in its piezoresistance. The other quasi-zero-dimensional nanostructures (i.e., OLC and CNH) initially have very high resistances of approximately 4 and 10 Ω, respectively. However, the resistance of these CNP drops considerably as the load increases, with both experiencing a reduction of approximately one order of magnitude. The comparison between the absolute and normalized resistance is important because it highlights the sensitivity of the piezoresistive behavior. Counterintuitively, higher resistances are more desirable because they require measurement equipment with lower resolutions. Additionally, higher slopes in load-resistance curves are also sought after because they imply higher responsiveness. According to these criteria, OLC and CNH are the most promising CNP. However, when considering the compression-to-displacement graphs from Figs. 5b, c and 6b, c, it is evident that stable displacement value per 1 N increment require loads above 6 N. The CNP with stable compression-to-displacement graphs (i.e., CB, GF to a certain extent, GNP for low loads, and CNT) do not exhibit as significant reduction in resistance during compression. Therefore, instrumentation with higher precision is necessary to accurately measure electrical resistance. CB may be a promising alternative due to its relatively high absolute resistance (in the ohm range) and a steeper load-resistance curve compared to one- and two-dimensional nanostructures (i.e., GF, GNP, CNT, and CNT-D).

Moreover, the methodology proposed by Tamai et al. to determine the predominant deformation mechanism in contacting surfaces was analogously employed in this study^40^. This evaluation was carried out to determine the validity of the methodology in conductive powders. The full range load-resistance curves (Fig. 11a) were linearly fitted to obtain the corresponding slope values, which are presented in Table 2. As shown in Table 2, the slope values coincide, to a certain extent, with the theoretical values corresponding to a predominance of elastic deformation (i.e., − 1/3 and − 2/3). Therefore, the slope analysis confirms that all CNP underwent elastic deformation within the vessel for the entire of the loading range evaluated. Furthermore, most of the resistance of the nanostructures originates predominantly from constriction resistance (slope ≈ − 1/3) except for OLC and CNH where film resistance dominates the system (slope ≈ − 2/3). Although CB is also a zero-dimensional nanostructures, film resistance is not the dominant resistance type, which could be due to the content of graphitic carbon present in this structure. Graphitic carbon may occupy the interstices within each nanoparticle, forming percolation paths that aid the motion of electrons within and between CB particles. Consequently, constriction resistance is more dominant. The other zero-dimensional nanostructures, however, are not as interconnected as CB but rather form individual clusters, contributing to an increased electrical resistance, which corresponds to the film resistance in this analogy. Therefore, constriction resistance is not as dominant in these CNP. Although this methodology is purely theoretical and idealized by nature, the experimental results demonstrated here confirm its validity and applicability—considering the parameters and assumptions outlined in this work.

**Table 2:** Slope value of linear fitting of load dependent resistance curves (plotted in double logarithmic scale), R-squared of linear fit between 0.94 and 0.99.

CNP	Slope	Dominant resistance type
CB	− 0.38 ± 0.01	Constriction
OLC	− 0.63 ± 0.04	Film
CNH	− 0.62 ± 0.01	Film
CNT	− 0.29 ± 0.01	Constriction
CNT-D	− 0.32 ± 0.01	Constriction
GF	− 0.38 ± 0.03	Constriction
GNP	− 0.35 ± 0.01	Constriction

## Conclusions

This study experimentally determined and discussed the piezoresistive performance of bulk carbon nanoparticles. Ohmic response, load-dependent contact resistance, and particle morphology were comprehensively characterized. The potential applicability of CNP due to their piezoresistive behavior is highlighted. It is shown that the morphology of the nanostructures plays a crucial role in their piezoresistive performance. Quasi-zero-dimensional nanostructures exhibit the highest absolute electrical resistance, as observed in CB and CNH. One-dimensional nanostructures, such as CNT and CNT-D, exhibit significantly lower electrical resistance than the zero-dimensional structures by approximately one order of magnitude, whereas two-dimensional structures present slightly lower electrical resistance than one-dimensional nanoparticles. OLC, in particular, fall between zero- and one-dimensional CNP in terms of electrical resistance.

The resistance of OLC decreases in a linear manner as the load increases (when plotted in a logarithmic scale), however the displacement fluctuates at low loads. Accordingly, the use of this nanoparticle for its piezoresistive performance must be limited to high load applications (above 6 N under the conditions of this study). Likewise, GNP also exhibit exceptional linearity. However, the compression-to-displacement relationship varies significantly for loads above 3 N. Therefore, the use of this nanoparticle should be limited to low load and relatively high current applications so as to minimize the resistance caused by capacitances (interlayer gap). GF on the other hand, should be limited to low and high load applications due to fluctuations in its compression-to-displacement relationship in mid-range loads. In relative terms, CNH presents the largest resistance reduction within the loading range (along with OLC), decreasing in a relatively linear manner as the load increases. Its compression-to-displacement relationship is exceptionally constant with minor fluctuations in the mid-range loads. Therefore, as with GF, its application should be limited to low and high load requirements. However, CNH possesses a larger ohmic range, albeit its electrical resistance is significantly higher. CB shows very promising results due to its consistency in its compression-to-displacement relationship in the mid-to-high loading range after the voids formed due to agglomeration are filled. The load-resistance curves are linear, and CB has a significant resistance reduction between 0.25 and 10 N. Moreover, CNT performed exceptionally well, having consistent compression-to-displacement relationship, linear load-resistance curves, and adequate overall resistance reduction. The latter is a significant advantage since instrumentation with lower resistance precision are required. Smaller agglomerate sizes did not benefit their piezoresistive performance. CNT-D’s electrical performance marginally improved compared to CNT. Its compression-to-displacement relationship, on the other hand, fluctuates throughout the entire loading range evaluated, tending to stabilize for high loads. Therefore, CNT-D should be utilized in very high load applications. Conversely, the reduction of agglomerate sizes (through dispersion) can be neglected since CNT in their agglomerated state are suitable for low, mid, and high load applications. Moreover, one- and two-dimensional nanostructures show relatively low electrical resistance at 10 N. Therefore, for sufficiently high loads, nanoparticle aggregates could be used as interconnection media between two terminals.

The results presented demonstrate the auspicious piezoresistive behavior of carbon nanostructures, emphasizing the importance of proper CNP selection based on intended applications. However, further research is necessary to gain a comprehensive understanding of their performance, efficiency, reliability, and limitations. Therefore, it is important to study the influence of atmospheric conditions (i.e., temperature and primarily humidity) and particle size, as well as loading conditions that exceed the ranges from this study. Additionally, while the measurements reported here showed exceptional repeatability, it is necessary to evaluate the CNPs’ performance in successive loading and unloading cycles in more detail.

### Supplementary Information

Supplementary Figures.

## Data Availability

The data required to support the present findings are present in the manuscript and supplementary information.
