# Supplementary material for: Evaluating the effect of unidirectional loading on the piezoresistive characteristics of carbon nanoparticles
Source: Sci Rep. 2024 Apr 22;14:9247. doi: 10.1038/s41598-024-59673-5 (PMC11035679; doi:10.1038/s41598-024-59673-5)
Supplement: Supplementary file 1 — Supplementary Figures. [file 41598_2024_59673_MOESM1_ESM.docx]

Submission ID: 88192325-4bc0-453a-8a2b-2288c65dc31a

Supplementary Information


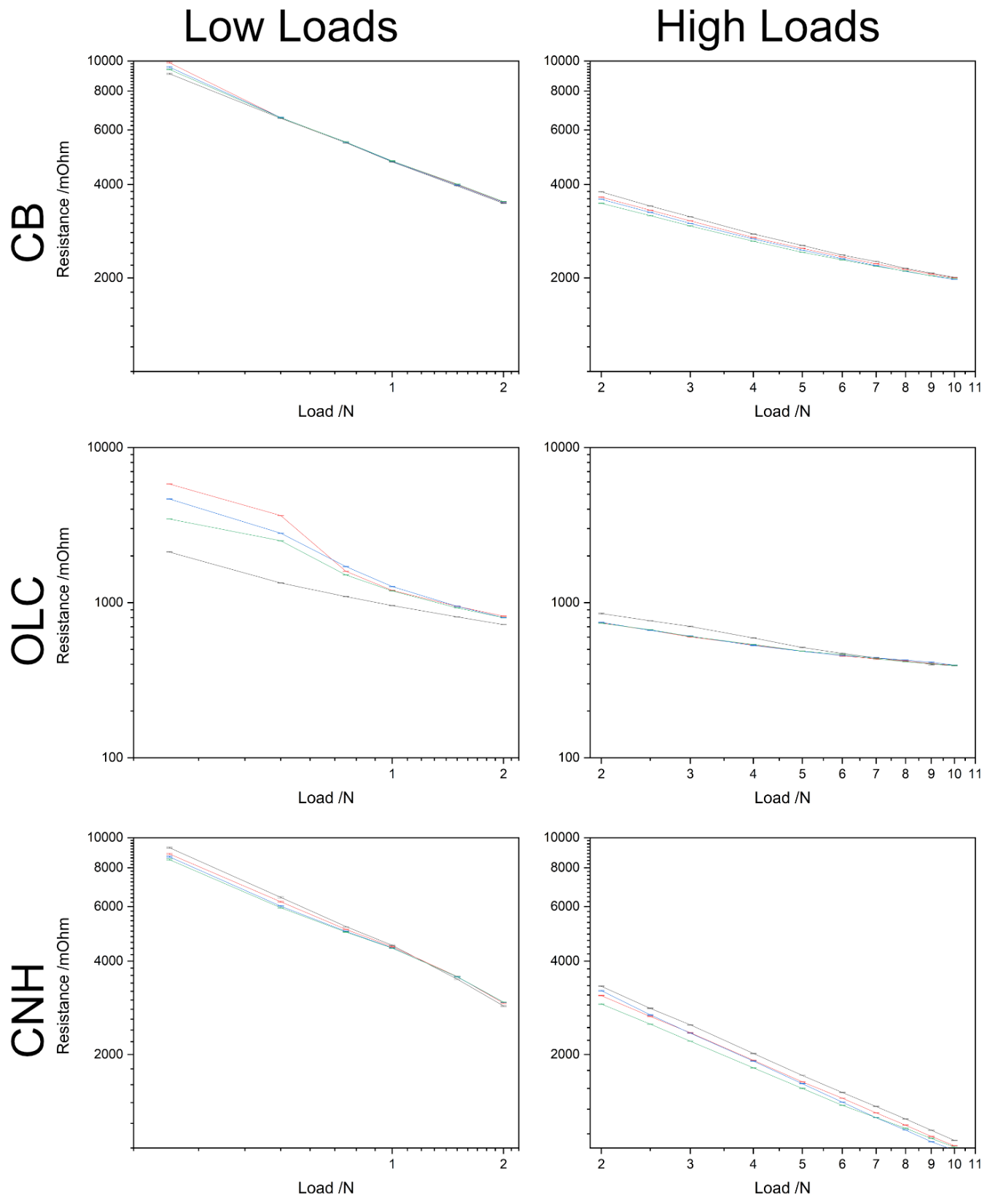


**Figure S1:** Multiple resistance curves of quasi-zero-dimensional CNP for low and high loading range highlighting the repeatability of the results.


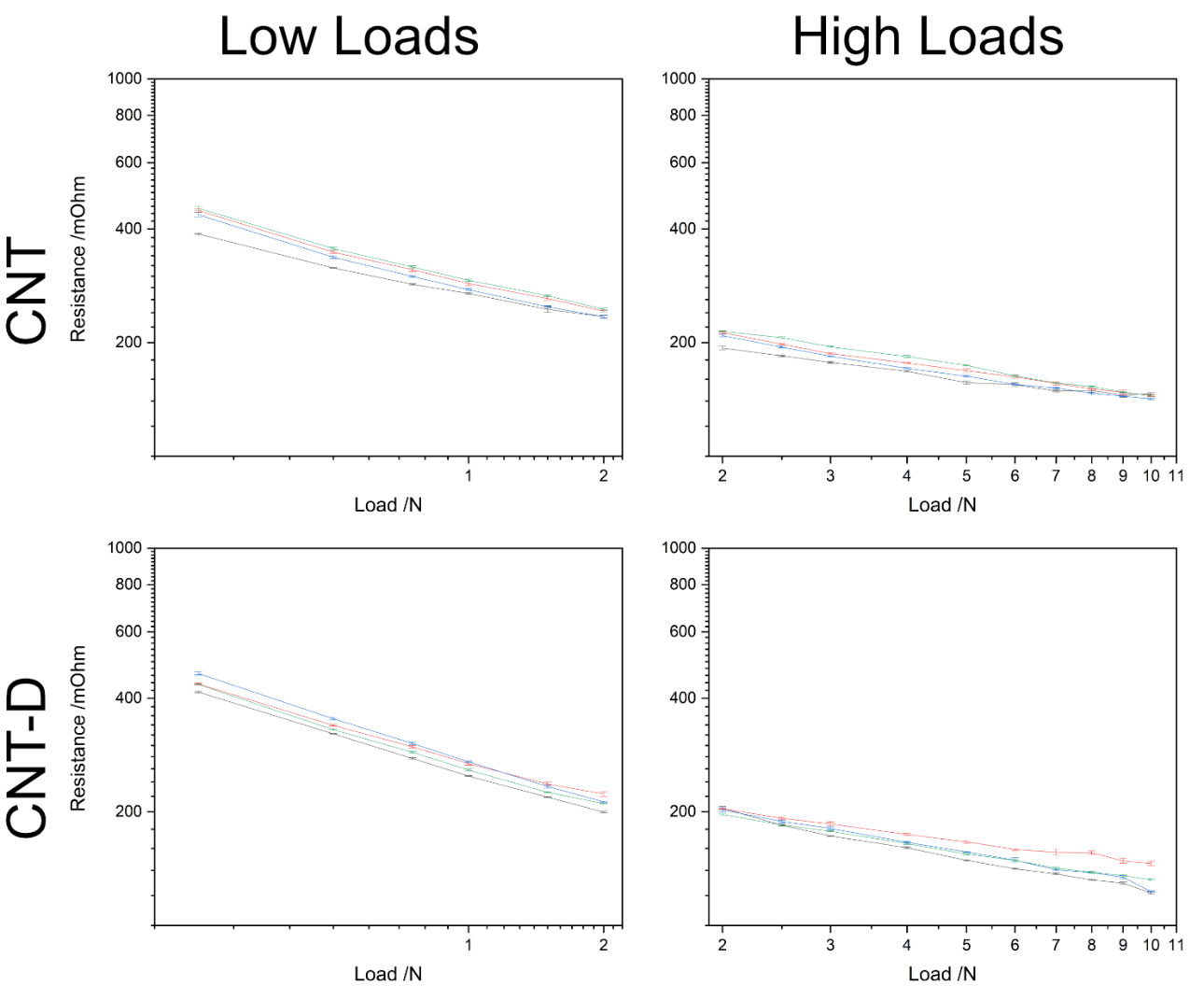


**Figure S2:** Multiple resistance curves of one-dimensional CNP for low and high loading range highlighting the repeatability of the results.


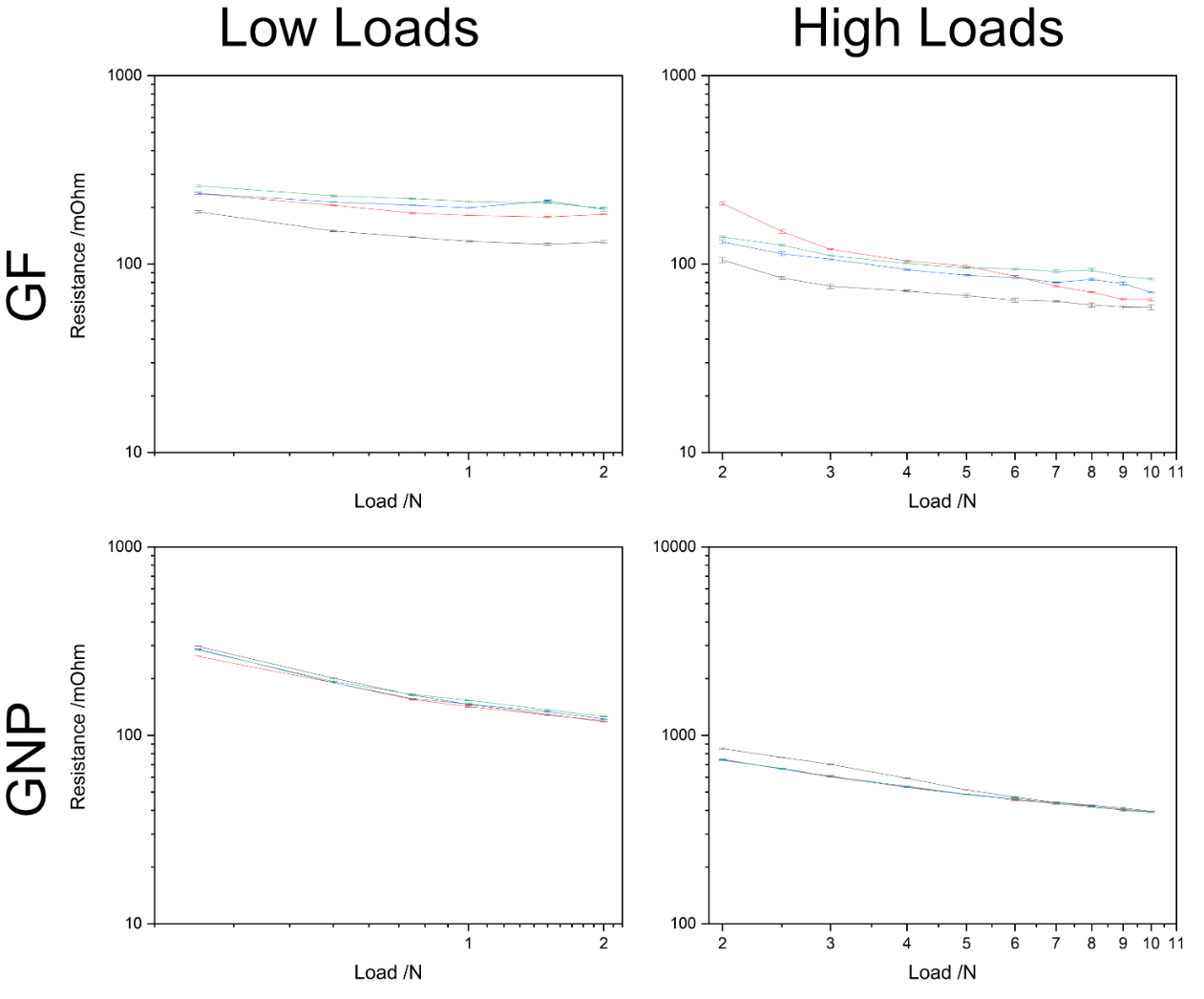


**Figure S3:** Multiple resistance curves of two-dimensional CNP for low and high loading range highlighting the repeatability of the results.

**Supplementary Figure legend:**

**Figure S1:** Multiple resistance curves of quasi-zero-dimensional CNP for low and high loading range highlighting the repeatability of the results.

**Figure S2:** Multiple resistance curves of one-dimensional CNP for low and high loading range highlighting the repeatability of the results.

**Figure S3:** Multiple resistance curves of two-dimensional CNP for low and high loading range highlighting the repeatability of the results.
